# Clinical Neuropathology Practice News 3-2012: the “ABC” in AD – revised and updated guideline for the neuropathologic assessment of Alzheimer’s disease 

**DOI:** 10.5414/NP300512

**Published:** 2012-04-18

**Authors:** Gabor G Kovacs, Ellen Gelpi

**Affiliations:** 1 Institute of Neurology, Medical University of Vienna, Vienna, Austria; 2 Neurological Tissue Bank of the Biobank-Hospital Clinic-IDIBAPS, Barcelona, Spain

**Keywords:** Alzheimer's disease, amyloid-beta, Tau, diagnostic criteria

## Abstract

The two major approaches for the neuropathological assessment of Alzheimer’s disease (AD) related pathology have been based on the assessment of neuritic plaques (CERAD) and neurofibrillary pathology (Braak and Braak). In 1997 these two approaches were integrated in the criteria and recommendations of the National Institute on Aging and the Reagan Institute Working group. Recently a new guideline has been published by the National Institute on Aging-Alzheimer’s Association. This new guideline recognizes the existence of a pre-clinical stage of AD as part of continuous neuropathological changes in the background of the disease process, and it fosters the assessment of amyloid-b phases in addition to neurofibrillary degeneration and neuritic plaques following an “ABC” score. Further, it suggests protocols for the neuropathological assessment of additional/concomitant neurodegenerative and vascular pathologies. Altogether, the new guideline responds to the need for an update of the existing “1997 criteria” for AD. Continued studies will have to assess the added value of the new approach and the influence of interlaboratory and/or methodological differences on the implementation of these new recommendations.

## Background 

In the past decades, neuropathological diagnosis of Alzheimer’s disease (AD) was based on the semiquantitative evaluation of I) extracellular neuritic plaques composed of amyloid-beta (Ab) surrounded by dystrophic neurites and II) on the distribution of neurofibrillary degeneration characterized by phospho-tau (pt) immunoreactive intraneuronal fibrillary deposits, together usually referred to as AD-related lesions. The two major approaches for the neuropathological assessment were the so-called CERAD criteria (Consortium to Establish a Registry for Alzheimer’s Disease) for neuritic plaque scoring [1] and the neurofibrillary pathology staging system proposed by Braak and Braak [2]. In 1997 these two approaches were integrated in the criteria and recommendations of the National Institute on Aging and the Reagan Institute Working group [3]. Presence of clinical diagnosis of dementia was an important aspect for classifying the level of likelihood of AD-related lesions being the substrate of the cognitive decline (“Thus, based on the pathological changes detected in the post-mortem brain alone (i.e., AD lesions), only probabilistic statements about the presence or absence of dementia can be made in a given patient.”) [3]. 

In recent years, excellent antibodies for immunohistochemical investigations have become available which disclosed concomitant deposition of neurodegeneration related proteins as frequent finding in dementing illnesses [4]. These scientific advances have led to an update of the Braak and Braak neurofibrillary staging system, which is currently based on the assessment of pt-immunoreactive neurofibrillary degeneration in routinely processed brain tissue [5, 6]. Beyond that, it has been shown that Ab deposition generally follows predictable phases involving particular brain areas [7]. These developments have paved the path for revising and updating the “1997 Criteria” for AD [3]. Indeed, a new guideline has been recently published [8] accompanied by an article on the application of these guidelines in neuropathology practice [9]. 

## What is new? 

The novelty of the guideline can be summarized as follows [8, 9]:

It recognizes the existence of a pre-clinical stage of AD as part of a continuum of neuropathological changes underlying the progression of disease;
It fosters the assessment of Ab phases in addition to the assessment of neurofibrillary degeneration and neuritic plaques following an “ABC” score;
It suggests protocols for the neuropathological assessment of concomitant Lewy body disease, vascular brain injury, hippocampal sclerosis, and TDP-43 pathology.

Altogether the guidelines recommend comprehensive standard approaches for the neuropathological assessment of AD-related pathology in post-mortem brain tissue including methodological aspects and clinico-pathologic correlations. 

## How to assess Alzheimer’s disease related lesions? The “ABC” score 

There are three main components related to AD pathology that need to be assessed (A, B and C; see below), and each component is assigned with 1 of 4 scores (0, 1, 2, 3): this is termed the ABC score. In a further step, these scores are combined and cases are identified as having high, intermediate or low level of AD neuropathological changes. These levels are ultimately correlated with the presence or absence of cognitive impairment, and with the presence/absence and extent of other diseases that might have contributed to the clinical deficits. 

## The “ABC” score 

### A (“A” for Amyloid)

It is recommended to assess the severity of Ab deposits based on the phase assessment described by Thal et al. in 2002 [7]. Since originally there are 5 phases described, score 1 includes Phases 1 + 2, Score 2 Phase 3, and Score 3 Phases 4 + 5. Score 0 indicates absence of Ab deposits. 

### B (“B” for Braak)

Neurofibrillary degeneration should be assessed based on the staging system described by Braak and Braak in 1991 [2] (based on silver stain) or 2006 [5] (based on pt – immunohistochemistry). As originally 6 stages have been described, Score 1 includes Stages I + II (or transentorhinal stage), Score 2 Stage III + IV (or limbic stage), and Score 3 Stages V + VI (or isocortical stage). Score 0 indicates absence of neurofibrillary pathology. 

### C (“C” for CERAD)

Finally, the evaluation of neuritic plaques is recommended based on the semiquantitative scoring system described by Mirra et al. in 1991 [1] (CERAD criteria): Score 1 refers to sparse, Score 2 to moderate and Score 3 to frequent neuritic plaques. Score 0 indicates absence of neuritic plaques. 

With regard to additional/concomitant pathologies it is recommended to assess neuronal alpha-synuclein (αS) pathology, and to classify it into 5 categories (modified McKeith criteria of DLB) [10], i.e. none, brainstem-predominant, limbic (transitional), neocortical, and amygdala-predominant; to describe the extent and type of vascular pathology; and to report the presence or absence of hippocampal sclerosis and TDP-43 pathology. 

## Conclusions 

The new guideline responds to the need for an update of the existing “1997 Criteria” for AD [3]. Continued studies will have to assess the added value of the new approach and the influence of interlaboratory and/or methodological differences on the implementation of these new recommendations (see: *Comment on the consensus recommendations for the postmortem neuropathological assessment of Alzheimer’s disease* in www.alzforum.org). 
